# Thermodynamic Evidence for Type II Porous Liquids

**DOI:** 10.1021/acs.iecr.3c01201

**Published:** 2023-07-11

**Authors:** Isaiah Borne, Kartik Saigal, Christopher W. Jones, Ryan P. Lively

**Affiliations:** School of Chemical & Biomolecular Engineering, Georgia Institute of Technology, Atlanta, Georgia 30332, United States

## Abstract

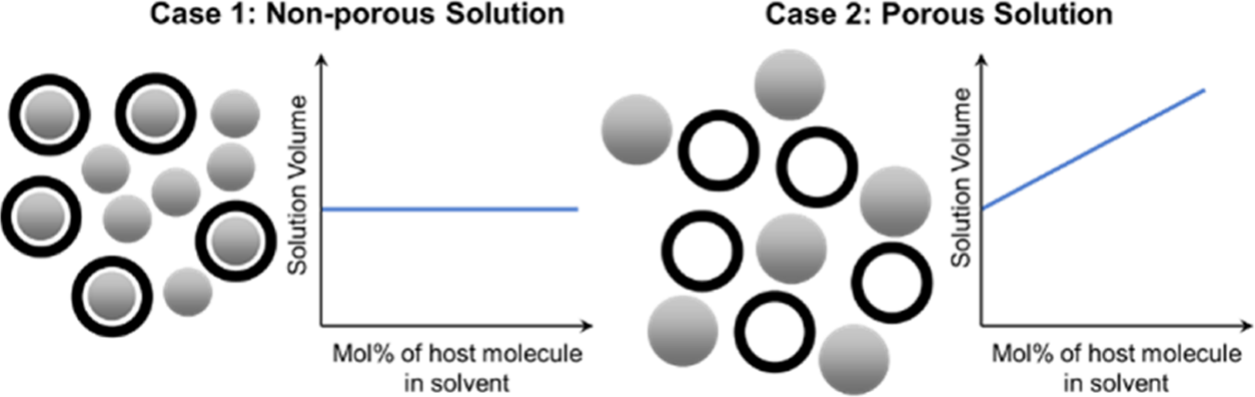

Porous liquids are an emerging class of microporous materials where intrinsic, stable porosity is imbued in a liquid material. Many porous liquids are prepared by dispersing porous solids in bulky solvents; these can be contrasted by the method of dissolving microporous molecules. We highlight the latter “Type II” porous liquids—which are stable thermodynamic solutions with demonstrable colligative properties. This feature significantly impacts the ultimate utility of the liquid for various end-use applications. We also describe a facile method for determining if a Type II porous liquid candidate is “porous” based on assessing the partial molar volume of the porous host molecule dissolved in the solvent by measuring the densities of candidate solutions. Conventional CO_2_ isotherms confirm the porosity of the porous liquids and corroborate the facile density method.

## Introduction

Porous liquids present an opportunity to combine the physical properties of flowing liquids with intrinsic microporosity usually associated with solids. The concept of porous liquids was proposed in 2007 by O’Reilly et al., who classified these materials into four different types of liquids for use in various applications.^1−8^ Type I porous liquids are neat liquids composed of a discrete porous material that maintains its microporosity in the fluid state.^9−14^ Type II porous liquids are composite materials developed by dissolving a discrete, rigid porous material in a solvent that is too large to enter the pores of the host.^15^ Type III porous liquids are defined as dispersions where an insoluble porous particle is dispersed in a solvent too large to enter the pores.^16^ Lastly, Type IV porous liquids are extended frameworks that maintain their porosity in the liquid state due to strong physical interactions.^17−19^

Type II porous liquids are solutions of microporous host molecules or materials dissolved in a sterically hindered solvent that cannot penetrate the host.^20−23^ The host materials are typically porous organic cages^24−27^ (POCs) or metal–organic polyhedral/coordination cages.^28−30^ They are discrete entities and soluble in various organic solvents, making them ideal host molecules to make Type II porous liquids. Type III porous liquids are microporous extended framework materials dispersed in sterically hindered solvents that cannot penetrate the microporous frameworks. MOF and zeolite particles dispersed throughout ionic liquids or other viscous solvents are common examples of Type III porous liquids classified as kinetically stable dispersions.^5,31−36^ Despite being the most investigated porous liquid, Type III porous liquids are not thermodynamically stable, and the amount of time these materials stay dispersed depends on the kinetic stability of the dispersion.

**Figure 1:**
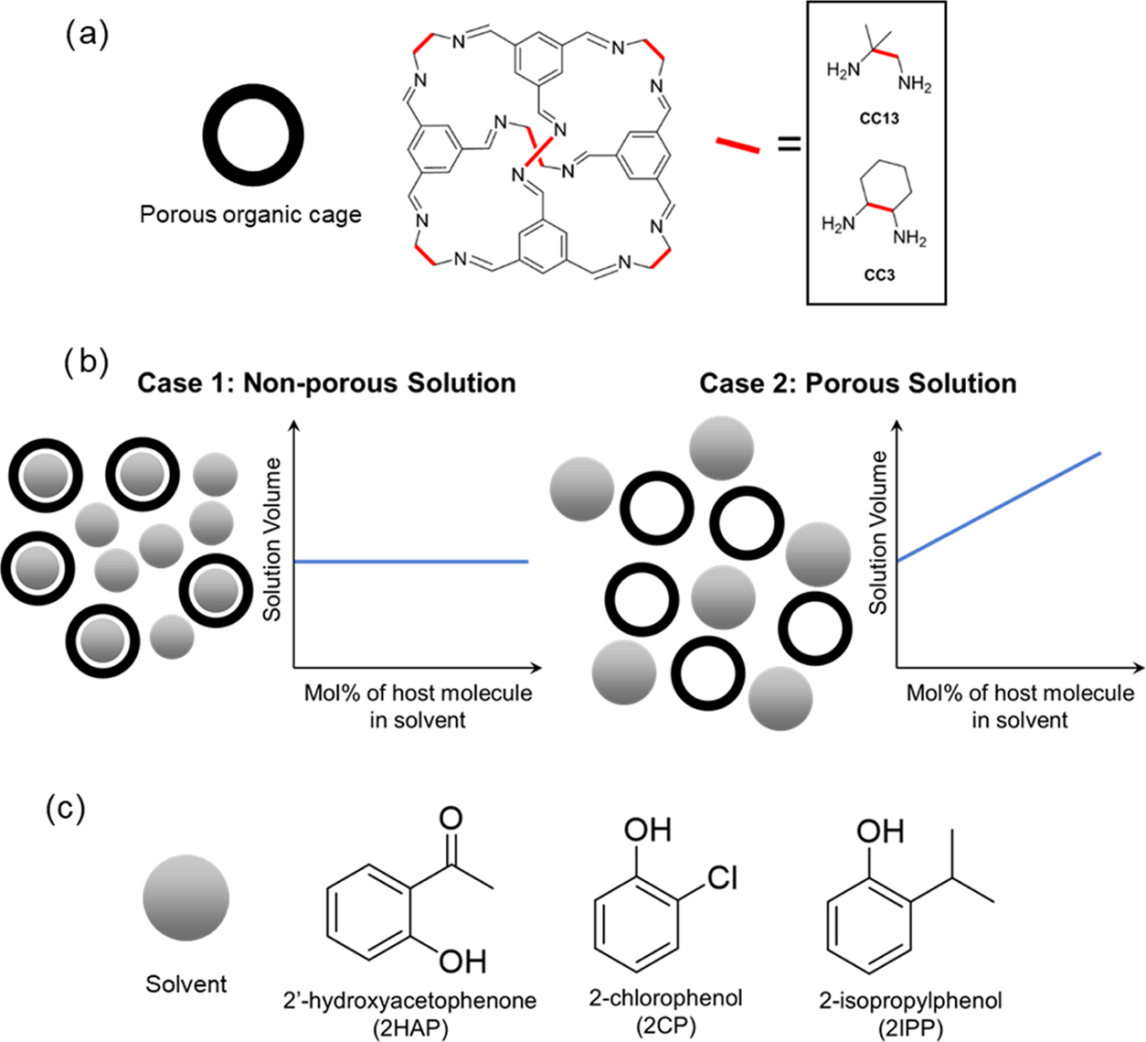
Porous organic cage structures, hypothesized solution volume trends for POCs in nonporous and porous solutions, and bulky solvents used for Type II porous liquids. (a) General chemical structure for [4 + 6] imine-based porous organic cages. The diamine linkers employed in the Schiff base reaction to develop CC13 and CC3 are highlighted (1-methylpropane-1,2-diamine for CC13 and 1,2-cyclohexanediamine for CC3). (b) Schematic describing expected solution volumes when adding porous organic cages to a solvent that is smaller than the pores of the cage (Case 1) and a solvent that is too large to enter the pores of the cage (Case 2). (c) Chemical structures of bulky organic solvents employed in this work.

On the other hand, if Type II porous liquids are truly thermodynamic solutions, they should remain stable at all times, especially in flowing conditions commonly envisioned for their various use cases. Thermodynamic solutions exhibit changes to colligative properties to the pure solvent simply as a function of the concentration of the porous molecule dissolved in the liquid regardless of the identity or nature of the porous molecule. These properties include vapor pressure, osmotic pressure, boiling point, and freezing point. In this work, the porous organic cage known as CC13 is used since it is highly soluble in various organic solvents (Figure 1a). We explore the freezing point of the porous liquids with increasing POC loading as an example colligative property. We contrast these experiments against dispersions composed of the POC known as CC3, which is insoluble in the solvents selected in this study.

Another key aspect of characterizing porous liquids is determining if a new porous solution candidate is truly porous. The state-of-the-art methods for characterizing the porosity of candidate porous liquids are through gas evolution tests, gas isotherms, or positron annihilation lifetime spectroscopy (PALS) experiments. These methods can be time-intensive, difficult to repeat, and require a significant amount of microporous material. A facile method for determining the porosity of porous liquids would significantly accelerate the development and implementation of porous liquids in relevant separations. We can use the partial molar volume (defined as the total change in solution volume with respect to the change in moles of one component of a solution, shown in eq 1) to determine if a liquid is porous or not.
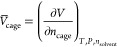
1In eq 1, *V̅*_cage_ represents the partial molar volume of the porous organic cage, *V* represents the total solution volume, *n*_cage_ stands for the number of moles of the porous organic cage, and *T*,*P*,*n*_solvent_, respectively, represent the temperature, pressure, and number of moles in the solvent.

We hypothesize that for nonporous liquids composed of small solvents (small solvents are solvents that can penetrate the pores of the cage), the partial molar volume of POCs will be small or negligible, whereas for porous liquids composed of bulky solvents (bulky solvents are solvents that cannot penetrate the pores of the cage), the partial molar volume of POCs will be larger, as shown in Figure 1b. To probe this hypothesis, we dissolved CC13 in solvents displayed in Figure 1c, which have been shown to create porous liquids (2′-hydroxyacetophenone, 2-chlorophenol, and 2-isopropylphenol). Previous works have shown that small solvents like chloroform and dichloromethane dissolve POCs like CC13 and CC3 and penetrate the pores of the POCs.^12,17^ This work shows that by measuring the density of POCs in various solvents, the partial molar volume of POCs in a solution can be calculated and confirm the porosity of candidate porous liquids in a facile and reliable manner. Decreases in density as POC solution concentration indicate successful development of Type II porous liquids, as suggested by previous work with Type III porous liquids.^37,38^

## Methods

### Materials

2′-Hydroxyacetophenone, 2-chlorophenol, and 2-isopropylphenol were purchased from Sigma-Aldrich. 1,3,5-Tricarbaldehydebenzene was purchased from Manchester Organics, and 1-methyl-1,2-diaminopropane was purchased from TCI Chemicals. Dichloromethane and chloroform were purchased from VWR. 1,2-Cyclohexanediamine (R,R) and trifluoroacetic acid were purchased from Sigma-Aldrich. Ultrahigh-purity CO_2_ was obtained from AirGas. All chemicals were used as received.

### CC13 Synthesis

CC13 was synthesized according to previous methods.^39^ Briefly, 1,2-diamino-2-methylpropane was dissolved in DCM and chilled with an ice water bath. A solution of 1,3,5-tricarbaldehydebenzene and DCM was added dropwise to the chilled 1,2-diamino-2-methylpropane solution. Once all of the tricarbaldehydebenzene solution was added, the ice bath was removed, and the reaction mixture was stirred for three days at room temperature. The resulting mixture was filtered, evaporated to a small volume through rotary evaporation, and then recrystallized with excess petroleum ether.

### CC3 Synthesis

CC3 was synthesized according to previous methods.^40^ Briefly, 1,2(R,R)-cyclohexanediamine was dissolved in DCM. Tricarbaldehydebenzene was also dissolved in DCM. The cyclohexanediamine solution was slowly added to the tricarbaldehyde solution. Upon complete addition of the cyclohexanediamine solution, a catalytic amount of trifluoroacetic acid was added to the solution, and the reaction was stirred at room temperature for three days. The solvent was removed from the reaction mixture via rotary evaporation, and the white solids were washed with ethyl acetate and dried in a vacuum oven.

### Characterization of Powder Porous Organic Cages

CO_2_ physisorption was performed on a Micromeritics ASAP 2020. Powders were degassed under vacuum (3–5 μm Hg) at 100 °C for 12 h before measurement. N_2_ physisorption at 77 K was conducted on a Belsorp MAX.

### Porous Liquid Isotherms

CO_2_ isotherms for the porous liquids were measured using a custom-built pressure decay cell. The liquid samples were loaded into the sample cell and initially saturated with He or N_2_ for 2 h. The inert gas in the sample chamber was quickly evacuated. CO_2_ was introduced to the sample cell, and the pressure decay was recorded over time. After the pressure in the sample cell was equilibrated (typically 4–6 h), the pressure of the sample cell was increased, and the pressure decay was again recorded. Data points were taken up to 5–6 bar. Schematics and additional details on these experiments are provided in the SI.

### Freezing Point Tests

A TA Q200 differential scanning calorimeter (DSC) was used to determine the freezing points of the porous liquids, dispersions, and their counterparts. The samples were rapidly frozen at −70 °C, then heated at 10 °C/min until −10 °C, and finally heated at 0.5 °C/min to 20 °C.

### Density Measurements

CC13 was dissolved in various organic solvents (DCM, chloroform, 2′-hydroxyacetophenone, 2-isopropylphenol, and 2-chlorophenol) at concentrations between 0 and 10 wt % cage. The density of these solutions was recorded using a 2 cm^3^ pycnometer. More specifically, the dry pycnometer was kept in a glass drying oven at 110 °C. The dry pycnometer was cooled to room temperature, then the mass was recorded three times, and the average mass was used as the empty weight for the density calculations. The solution was then added to the pycnometer until the pycnometer was full. The cap of the pycnometer was put on and then any excess solvent that was pushed out of the pycnometer was wiped off. The pycnometer filled with solution was placed on the scale, and the mass was measured three separate times. The mass of the empty pycnometer was subtracted from the mass of the full pycnometer to get the mass of the solution. The mass was then divided by the volume of the pycnometer (2 cm^3^) to get the resulting density of the solution.

### Partial Molar Volume Measurements

The partial molar volume of the cage in a solution is defined as the change in volume of a solution as a specified amount of cage is added while holding all other variables constant. The equation for partial molar volume is shown in eq 1, where *V̅* is the partial molar volume, *V* is the volume of the system, *n* represents moles, *T* is temperature, and *P* is pressure.

The partial molar volume of a solute in a solvent can be related to the density, similar to previous works.^41,42^ By starting with the partial molar volumes of the solvent (1) and cage (2), we can relate the partial molar volume of the cage in solution to the density of the solution as shown in eq 2.
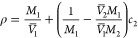
2In eq 2 (and all subsequent equations), ρ stands for the solution density, *M* represents a compound’s molar mass, and *c* is the concentration of the porous organic cage dissolved in the solvent.

The partial molar volume of the solvent and the cage can be determined by plotting the density as a function of the cage concentration and calculating the slope and y-intercept of eq 2. Equation 2 is valid for dilute solutions. For highly concentrated solutions, partial molal volumes can be used as shown in previous work.^43^ The detailed derivation is shown in the Supporting Information.

## Results and Discussion

### Freezing Point Analysis through DSC

**Figure 2:**
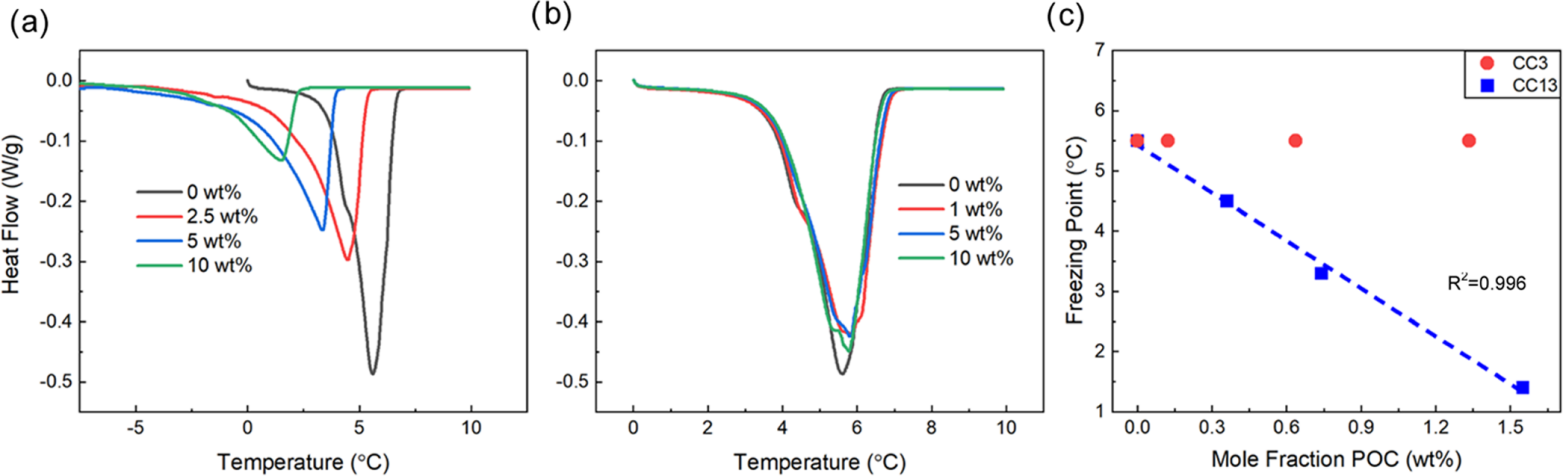
DSC thermograms showing melting point dependence on weight loading of (a) CC13 dissolved in 2HAP and (b) CC3 dispersed in 2HAP. (c) Freezing point of CC13 solutions and CC3 dispersions plotted versus mole fraction of POC in the solution/dispersion.

The classification framework for porous liquids, specifically the difference between Type II and Type III porous liquids, has not been rigorously experimentally tested. All solutions have colligative properties or physical changes that result from adding a solute to a solvent. One colligative property is freezing point depression, where the freezing point of a solvent decreases due to the addition of a solute. It is straightforward to use a DSC to record the freezing/melting point of solutions of CC13 in 2′-hydroxyacetophenone. This pair was chosen because the ligands used in CC13 have been used to create various porous organic cages for Type II porous liquids, and 2′-hydroxyacetophenone has been employed as a solvent capable of dissolving porous organic cages and creating porous liquids.^20^Figure 2 shows the thermograms for solutions of CC13 dissolved in 2′-hydroxyacetophenone and slurries of CC3 in 2′-hydroxyacetophenone. When observing the thermograms for the CC13 solutions, there is a clear depression of the freezing point as the weight loading of CC13 in the solution increases (up to 10 wt % or 1.6 mol %). The observed change in freezing point was larger than expected, as shown in Table S1, but Figure 2c shows the freezing point depression correlates very well with the mole fraction of POC in the solution. The freezing point depression exhibited by the CC13 and 2′-hydroxyacetophenone solution clearly shows that the mixture creates a thermodynamically stable solution.

A control experiment was conducted using CC3 and 2′-hydroxyacetophenone slurries because a slurry or dispersion should not exhibit the same colligative properties as a solution. For CC3, the melting point of the dispersions does not drop even up to a weight loading of 10 wt % (1.3 mol %) porous organic cage. There are shoulders introduced on the melting curves for the dispersions that are not present for the pure 2′-hydroxyacetophenone. Those shoulders may represent a low-temperature melting peak or a phase transition of the cages as the solvent is melting. A control DSC experiment was conducted on pure CC3 to determine if the shoulders were caused by some phase transition of the cage at those temperatures. The heat flow in the relevant temperature range exhibited a slight, linear decrease and showed no evidence of phase transition identifiable via DSC (thermogram is shown in SI). Even with the shoulders present, it is clear that CC3 dispersions do not exhibit a change in this colligative property, unlike the CC13 and 2′-hydroxyacetophenone solutions. Over the course of hours, the dispersed CC3 particles would settle and agglomerate, further supporting that CC3 and 2′-hydroxyacetophenone create a thermodynamically unstable dispersion. However, understanding the degree to which microporous materials in Type III porous liquids settle and deposit while flowing is important to determine which type of porous liquid is better suited for industrial use. More details on the importance of settling in Type III porous liquids are discussed in the SI.

### Partial Molar Volume and Density Measurements

**Figure 3:**
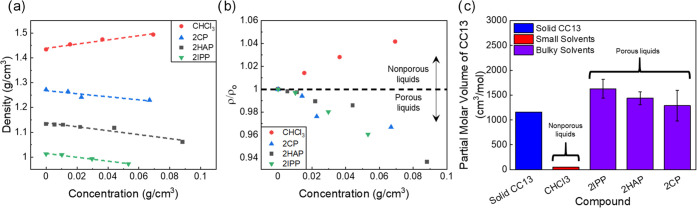
(a) Density of solutions composed of CC13 dissolved in various large solvents (2′-hydroxyacetophenone, 2-chlorophenol, and 2-isopropylphenol) and small solvent (chloroform) vs the concentration of CC13 dissolved in those solvents. (b) Normalized densities of CC13 solutions in various solvents. (c) Partial molar volumes of CC13 dissolved in various solvents as calculated by eq 2 compared to the molar volume of solid CC13.

CC13 solutions of varying concentrations (0–10 wt %) were created using bulky solvents like 2′-hydroxyacetophenone or small solvents that can enter the pores of the cage, like chloroform. We measured the densities of the solutions to test our hypothesis outlined in Figure 1b. For all solvents, the maximum weight loading of CC13 was set to 10 weight percent to avoid issues with gelation and inaccurate density calculations resulting from the gel. Figure 3a,b shows the densities of CC13 dissolved in 2′-hydroxyacetophenone, 2-chlorophenol, 2-isopropylphenol, and chloroform. CC13 dissolved in chloroform should align with case 1 in Figure 1b. A key observation for the density data for chloroform solutions was that as the cage concentration in the solution increased, the solution density also increased. This observation supported the idea that the small solvents fill the pores of the cage. For CHCl_3_, when the density plots were converted to partial molar volumes, the partial molar volumes of CC13 dissolved in the small solvents were positive. Specifically, for CC13 dissolved in CHCl_3_, the partial molar volume of CC13 was ∼50 cm^3^/mol. However, the partial molar volume of the cages in solution for the small solvents was significantly lower than the molar volume of solid CC13 (1160 cm^3^/mol), suggesting that the small solvents fill the CC13 pores.

Conversely, for the CC13 and 2′-hydroxyacetophenone solutions, the density decreased as the weight loading of CC13 increased (Figure 3a,b). This initial observation corroborated the idea that the large solvent cannot fill the pore of the cage and suggests that the partial molar volume of the cage dissolved in 2′-hydroxyacetophenone is similar to that of the solid cage. When the density of the CC13 solution was converted to partial molar volume, the results aligned with the original hypothesis. The molar volume of the solid CC13 is about 1160 cm^3^/mol (calculated from single-crystal data),^39^ and the partial molar volume of the dissolved CC13 in 2′-hydroxyacetophenone was approximately 1440 cm^3^/mol (Figure 3c). We tested two more solvents that create porous solutions to demonstrate that this method can be generalized to solvents other than 2′-hydroxyacetophenone. The density results for 2-chlorophenol and 2-isopropylphenol are also shown in Figure 3a,b. In previous work, the authors confirmed that CC13 dissolved in 2-chlorophenol and 2-isopropylphenol creates porous liquids.^44^ The porous solution density for both phenolic compounds decreased as the concentration of CC13 was increased. This decrease in density resulted in CC13 partial molar volumes of 1630 and 1290 cm^3^/mol CC13 in 2IPP and 2CP, respectively. Further analysis of the solution density in Figure 3b showed similar changes in the solution density for each bulky solvent. The normalized data supported the idea that CC13 similarly excludes the solvents and yields a porous liquid in each case. It is important to note that this method of measuring the density to calculate the partial molar volume of the porous solute in the solvent should only be applied to Type II porous liquids. Decreases in expected density have been observed for Type III porous liquids,^37,38^ and this behavior is still a qualitative indication of porosity. However, in a Type III porous liquid, the porous material is suspended and not dissolved to make a homogeneous solution, so the following partial molar volume calculations will not be physically relevant for a Type III porous liquid.

An extension of the porous solution density results is predicting the available pore volume of the porous organic cages while dissolved using the results in Figure 3c. When measuring the partial molar volume of the POCs when dissolved in a bulky solvent, the measured value corresponds to the skeletal volume of the cage and the intrinsic pore cavity. So, the partial molar volume of the cage dissolved in a bulky solvent should be a valid, rough estimate of the cage’s accessible pore volume in the dissolved state. Although this approach is not an absolute measure of the pore volume, like N_2_ physisorption at 77 K for porous solids, it can be used as an estimate to understand qualitative trends in the accessible pore volume of dissolved cages, as shown in Table 1. This table shows the calculated accessible pore volume of the cage in the porous liquid. The percent error was calculated using the known molar volume of CC13 from N_2_ physisorption as the reference point. The percent error for the accessible pore volume of CC13 in the dissolved state compared to the solid state was between 11 and 41%. Although the error was as high as 41% for the porous liquids, the N_2_ physisorption at 77 K isotherms on CC13β from previous work measured a pore volume that is 55% higher than the pore volume calculated computationally from single-crystal X-ray data (the higher pore volume calculated by N_2_ physisorption at 77 K is attributed to the extrinsic porosity of the CC13β crystal structure). The accessible pore volume calculated via the solution density seems reasonable since those values fall between pore volumes calculated computationally and measured via N_2_ physisorption at 77 K. It seems that the partial molar volumes extracted from the solution density measurements overestimate the accessible pore volume of CC13. The overestimate is probably due to the cages’ partial molar volume, which includes the cages’ skeletal framework.

**Table 1:** Estimated Pore Volume of CC13 Dissolved in Bulky Solvents Compared to Computational Measurements and N_2_ Physisorption

solvent	“accessible pore volume” (cm^3^/g)	% error between accessible and actual pore volume
CC13 (computational calculations)	1.21	
CC13β (N_2_ physisorption)	1.88	55
2IPP	1.69	40
2HAP	1.49	23
2CP	1.34	11

### Porous Liquid Isotherms

**Figure 4:**
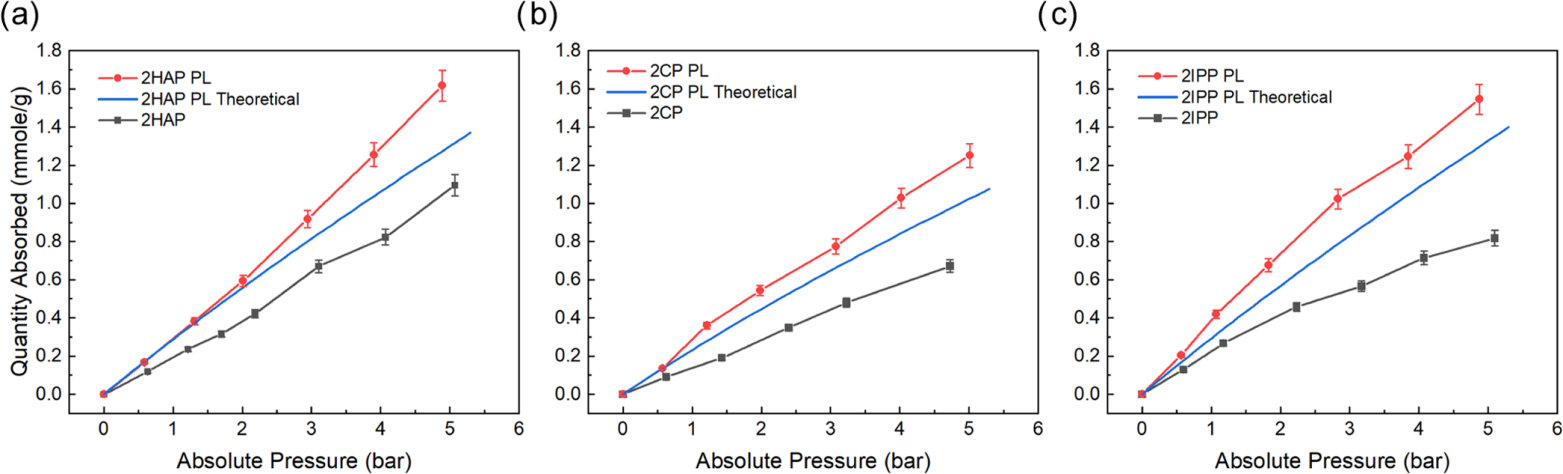
CO_2_ isotherms at 30 °C for various liquids: (a) 2′-hydroxyacetophenone and 10 wt % CC13 in 2′-hydroxyacetophenone, (b) 2-chlorophenol and 10 wt % CC13 dissolved in 2-chlorophenol, and (c) 2-isopropylphenol and 9 wt % CC13 dissolved in 2-isopropylphenol. Red circles represent experimental porous liquid isotherms, gray squares represent pure solvent isotherms, and solid blue lines represent weighted average isotherms for CC13 and pure solvent.

Solutions of CC13 (or ASPOCs using CC13 ligands) and 2′-hydroxyacetophenone, 2-chlorophenol, and 2-isopropylphenol have been shown to be porous in previous work through gas evolution experiments. CO_2_ isotherms at 30 °C were taken for 8–10 wt % solutions of CC13 dissolved in three different solvents: 2′-hydroxyacetophenone, 2-chlorophenol, and 2-isopropylphenol using a custom-built pressure decay cell. Although CC13 can be dissolved to much higher weight loadings in these solvents, we kept the weight concentration low to observe the gas capacity for low-viscosity liquids and not high-viscosity liquids or even gels that have been reported at higher concentrations. The resulting isotherms are shown in Figure 4. The pure solvents show linear CO_2_ uptake as a function of pressure, which was expected and agreed with Henry’s law. 2′-Hydroxyacetophenone showed the highest uptake at 1.1 mmol/g at 5.1 bar, followed by 2-isopropylphenol with an uptake of 0.81 mmol/g at 5.1 bar, and 2-chlorophenol exhibited the lowest uptake of 0.67 mmol/g at 4.7 bar.

There is a clear increase in CO_2_ gas capacity for each solvent compared to the pure solvent for the porous liquid, especially at lower pressures (between 0 and 2 bar). The increased gas capacity comes from the POC’s intrinsic microporosity, which is captured by the partial molar volume experiments highlighted earlier in this work. Even with only 10% weight loading of CC13 in 2′-hydroxyacetophenone, there were noticeable increases in CO_2_ uptake. The CC13/2′-hydroxyacetophenone porous liquid shows a 40–50% increase in the CO_2_ uptake at all pressures compared to the CO_2_ uptake of pure 2′-hydroxyacetophenone. At 4.9 bar, the porous liquid absorbs 1.62 mmol/g compared to 1.1 mmol/g at 5.1 bar for the pure solvent. The increase in gas capacity across the entire pressure spectrum suggests that the cages are actively adsorbing CO_2_ not just at low/ambient pressures but also at higher pressures as well. Similar results are seen for 2-isopropylphenol and 2-chlorophenol. At 10% weight loading of CC13 in 2-chlorophenol, the CO_2_ uptake almost doubled relative to the pure solvent. At 5 bar, the porous liquid with 2-chlorophenol had an uptake of 1.25 mmol/g. Likewise, for CC13 dissolved in 2-isopropylphenol, the resulting porous liquid’s CO_2_ capacity increased by about 100% relative to the pure solvent. The increase in gas capacity for the porous liquids in this study relative to the pure solvents is consistent with previous gas uptake results for Type II porous liquids. Theoretical CO_2_ isotherms were calculated by taking the weighted average of the CO_2_ isotherms for CC13 and the pure solvents (CO_2_ isotherm for CC13 up to 5 bar in Table S4), and the calculated isotherms agree well with the experimental results. The experimental isotherms are 10–15% higher than that of the weighted average isotherms, suggesting some synergistic impact between the cages and the solvent on the overall CO_2_ uptake for the porous liquid. This synergistic effect in Type II porous liquids has been reported in previous work.^38,45^ However, for porous liquids developed from 2′-hydroxyacetophenone, the synergistic effect on CO_2_ uptake is only observed from 2 to 5 bar. Other methods outside of the scope of this work may be useful for exploring this observation. The results shown in Figures 3 and 4 suggest that measuring the solution density of porous liquid candidates is a quick, facile, and reliable method for determining the porosity of a liquid.

## Conclusions

Here, we provide experimental evidence that Type II porous liquids behave as thermodynamic solutions. CC13 was used as our microporous host due to its high solubility and was confirmed to create Type II porous solutions by DSC measurements. DSC thermograms confirmed that CC13 does dissolve in 2′-hydroxyacetophenone since the colligative properties of the solvent change when adding CC13 to the solvent. The rate of change of the freezing point depression was consistent with the weight loading of CC13 added to the solvent. As a negative control, CC3 was dispersed in 2′-hydroxyacetophenone; as expected, that dispersion did not exhibit freezing point depression. The DSC measurements on the solution and dispersion differentiate between Type II and Type III porous liquids on a thermodynamic basis by analyzing the change in colligative properties.

Lastly, a simple method of measuring solution densities of porous organic cages and converting that value into partial molar volumes leads to much faster confirmation of the porosity of candidate porous liquids. Since bulky solvents cannot penetrate the pores of the host material in porous liquids, a porous liquid should have a lower density than a pure solvent. The density method confirmed the porosity of CC13 dissolved in 2′-hydroxyacetophenone, 2-chlorophenol, and 2-IPP, which have all been shown to be porous liquids in previous work. Isotherms were collected for the porous liquids to support the claim that solution densities can be used to calculate partial molar volumes of cages in solution and quickly determine whether a liquid is porous. Experimental absorption isotherms and critical deposition velocity analyses suggest that Type II porous liquids are interesting candidate materials for industrial gas separations.

## Supporting Information Available

CO_2_ isotherms for solvents and porous liquids with pressure decay cell method, raw freezing point depression data, CC13 DSC thermogram, critical deposition velocity of Type III porous liquids discussion, MOF densities table, critical deposition velocity figures, CC13 pressure decay cell isotherm, and raw data for all CO_2_ pressure decay cell isotherms (PDF)
