# Supplementary material for: Thermodynamic Evidence for Type II Porous Liquids
Source: Ind Eng Chem Res. 2023 Jul 11;62(29):11689–96. doi: 10.1021/acs.iecr.3c01201 (PMC10375470; doi:10.1021/acs.iecr.3c01201)
Supplement: Supplementary file 1 — ie3c01201_si_001.pdf [file ie3c01201_si_001.pdf]

Supporting Information

Thermodynamic Evidence for Type II Porous Liquids

Isaiah Borne, Kartik Saigal, Christopher W. Jones, and Ryan P. Lively*

School of Chemical and Biomolecular Engineering, Georgia Institute of Technology, Atlanta, GA 30332, USA

*Corresponding Authors: ryan.lively@chbe.gatech.edu

Table of Contents

Methods…………………………………………………………………………………...3

Freezing Point Depression Data…………………………………………………………7

CC3 DSC Thermogram……………………………………………………………………7

Critical Deposition Velocity Discussion…………………………………………………8

MOF Densities………………………………………………………………………………9

Critical Deposition Velocity Plots………………………………………………………...10

CC13 Isotherm……………..…………………………………………………………...11

Isotherm Raw Data…………………………………………………………………..12

References…………………………………………………………………………….13

CO_2 Isotherms for Porous Liquids with Pressure Decay Cell:

A schematic showing the pressure decay cell used to measure the porous liquid isotherms is shown in **Figure S1**. The experimental procedure is as follows. First, the sample container (a 500 μm filter element) was soaked in acetone and sonicated for 5 minutes to fully clean it. The sample container was then placed in a glassware drying oven at 110 °C for about 10 minutes to dry off the residual acetone. Next, the mass of the dry sample container was measured and recorded. The mass of a small amount of aluminum foil meant to cover the top of the sample container was measured and recorded along with a thin copper wire. Finally, the liquid samples (~0.1 mL) were loaded into the sample container, covered with aluminum which was secured with thin copper wire to secure the aluminum foil. The mass of this full system was recorded and through back calculation the mass of the liquid could be calculated. The covered sample container was then placed into the sample cell and the oil bath was set to the analysis temperature. The liquid sample was initially saturated with helium for 1-2 hours to degas the porous liquid of any pre-adsorbed CO_2 or other gases. Typically, porous solids are degassed under high temperature and high vacuum, but our solvents are volatile; thus, traditional degassing methods would evaporate all of the solvents. To saturate the porous liquid with helium, the dosing chamber and manifold was evacuated quickly (~ 5 minutes) and then pressurized with helium to about 20 psi. The valve connecting the sample cell to the dosing chamber was closed and the system was left static for 1-2 hours. The helium in the sample chamber was then evacuated quickly again (~1-2 minutes). CO_2 was introduced to the dosing chamber, allowed to thermally equilibrate for 2-5 minutes (or until the pressure was stable), then the valve connecting the dosing chamber and the sample cell was opened for 2 seconds then closed. The pressure decay in the sample cell was recorded over time. After the pressure in the sample cell equilibrated (typically 4-6 hours) the pressure of the sample cell was increased and the pressure decay was again recorded until 5-6 bar was reached. The pressure decay for each sorption step was related to the number of moles of CO_2 that entered the porous liquid or neat solvent via the ideal gas law. At pressures above 1 bar compressibility factors were applied to the ideal gas law to correct for non-idealities.

Partial Molar Volume from Density Derivation

By starting with the partial molar volume of the solution in equation S1, we can derive a connection between the total solution volume and the constituent materials volume.

$$V = n_1\overline{V}_1 + n_2\overline{V}_2 \tag{S1}$$

First, divide the entire expression in equation S1 by the total number of moles in the system, where x represents the mole fraction of species 1 or 2.

$$\hat{V} = x_1 \cdot \overline{V}_1 + x_2 \cdot \overline{V}_2 \tag{S2}$$

Then divide equation S2 by the molar volume of the entire solution.

$$1 = \frac{x_1}{\hat{V}} \cdot \overline{V}_1 + \frac{x_2}{\hat{V}} \cdot \overline{V}_2 \tag{S3}$$

The mole fraction of a species in solution can be related to the mass fraction via equation S4. The relation in equation S4 can be substituted into equation S3 to obtain equation S5. In equation S4, w represents the weight fraction of species 1 or 2, ρ shows the density of the solution, and M is the molar mass of a species.

$$\frac{x_i}{\hat{V}} = \frac{\rho w_i}{M_i} \tag{S4}$$

$$1 = \frac{\rho}{M_1}(1 - w_2)\overline{V}_1 + \frac{\rho w_2}{M_2}\overline{V}_2 \tag{S5}$$

Lastly, the relation between the weight fraction, density, and concentration, c, shown in equation S6 can be substituted into equation S5 and rearranged to get equation 2, which relates density to the concentration of the cage in the solvent through a linear relationship.

$$w = \frac{c}{\rho} \tag{S6}$$

Critical deposition velocity (CDV) calculations

Type III porous liquids differ from Type II porous liquids in that they are dispersions instead of solutions. Type III porous liquids can potentially deposit microporous frameworks throughout operating units, which can lead to safety and economic concerns. The critical deposition velocity of potential and developed Type III porous liquids or porous dispersions on separations processes were calculated to understand the impact of Type III porous liquids or porous dispersions. The critical deposition velocity is the speed at which a slurry or dispersion must be maintained to prevent particles from depositing while flowing in a horizontal pipe.[1] The Thomson velocity has been applied to particles under 100 μm diameter particles and was used to calculate the CDV of MOF slurries in different solvents (Equation S7).[2] The densities of the MOFs were calculated using the Mercury software.[3-10]

$$v_T = 9\left(\frac{g\mu_f(S-1)}{\rho_f}\right)^{0.37}\left(\frac{D\rho_f}{\mu_f}\right)^{0.11} \quad (S7)$$

In equation 3, g represents the acceleration due to gravity, μ_f is the viscosity of the carrier fluid (for this correlation, it is assumed to be the viscosity of water, methanol, 2'-hydroxyacetophenone, or sesame oil), S is the ratio of the density of the solid to the density of the carrier fluid, D represents the diameter of the pipe, and lastly ρ_f represents the density of the carrier fluid. The Thomson critical deposition velocity is typically used as a lower bound value.

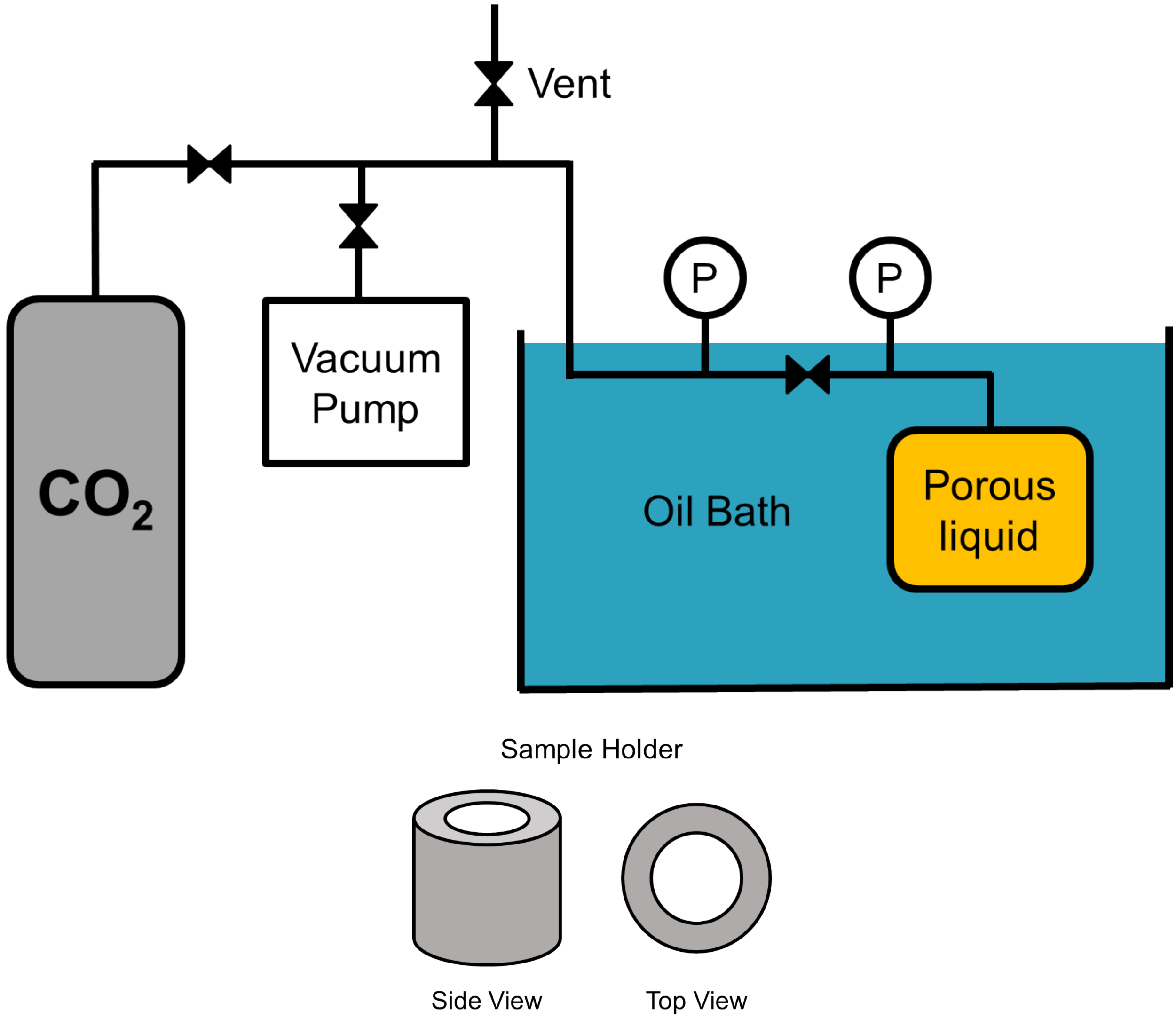

Figure S1: Schematic outlining design of pressure decay cell used to obtain gas isotherms for porous liquids and neat solvents (top), drawing of sample holder used to hold the liquids (bottom)

Table S1: CC13-2HAP Freezing Point Depression Data

CC13 weight loading (wt%)	Experimental ΔT_m (°C)	Theoretical ΔT_m (°C) ($i = 1$)	Theoretical ΔT_m (°C) ($i = 5$)
**2.5**	**1.0**	**0.18**	**0.92**
**5**	**2.2**	**0.38**	**1.88**
**10**	**4.1**	**0.79**	**3.97**

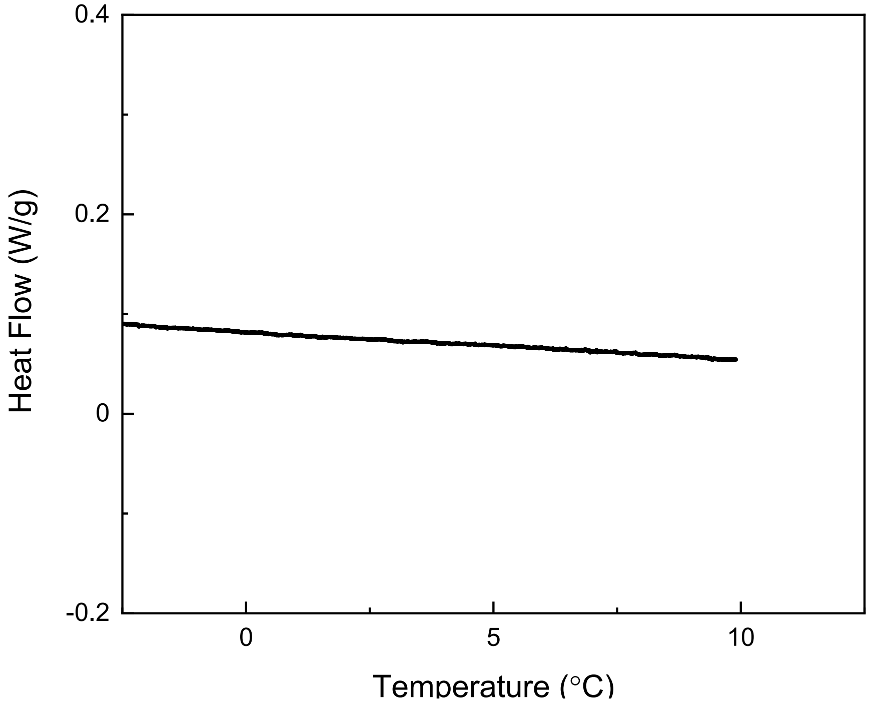

Figure S2: DSC thermogram of pure CC3

Critical Deposition Velocity of Type III Porous Liquids

The critical deposition velocity is defined as the speed at which a slurry or dispersion must be maintained to prevent particles from depositing while flowing in a horizontal pipe. In industrial gas absorption separations using solvents, it is preferred to have fluid velocities around 1-4 m/s, depending on the pipe diameter.[11, 12] Above these flow velocities, there can be safety concerns, including erosion and critical failure of the pipes. If Type III porous liquids are to be useful in the context of gas separations, then the deposition velocities of the particles must be below that range to avoid particle accumulation in the process units. However, upon analysis of the critical deposition velocities for various porous liquids, the critical deposition velocities for the Type III porous liquids are potentially much higher than 1-4 m/s, posing process complications. The trends in critical deposition velocity for dispersions as a function of various parameters are shown in **Figure S3**. According to the Thomson equation, the critical deposition velocities increase when using high viscosity fluids and high density particles. **Figure S3** highlights that for porous dispersions, it could be difficult to find a stable dispersion with a critical deposition velocity that allows for safe operation**.** It is important to note that various factors can impact the actual critical deposition velocity of Type III porous liquids. Specifically, the attractive forces between the solvent and host material could lead to a more stable dispersion or direct surface engineering of the porous solid, which could also enhance these interactions.

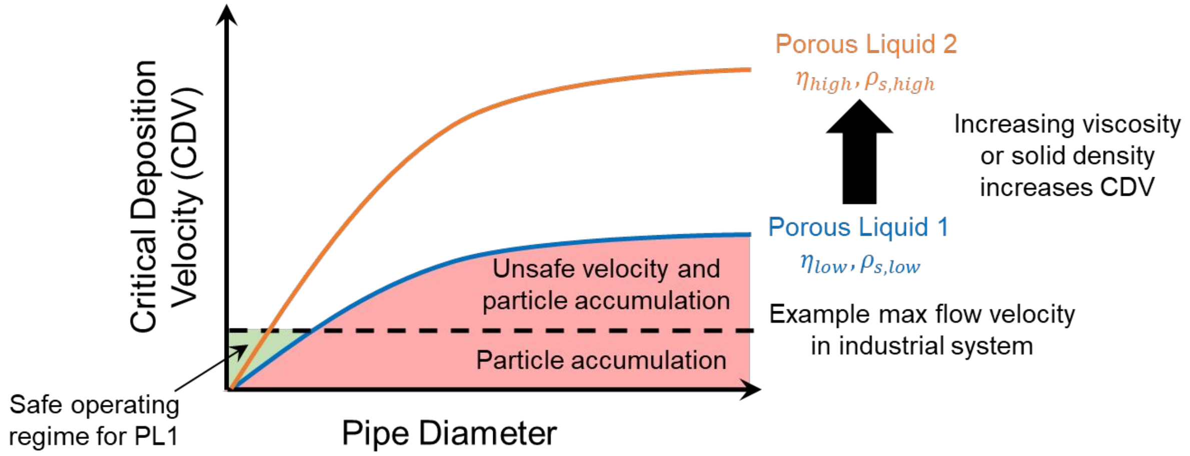

Figure S3: Qualitative plot of critical deposition velocity trends for slurry transport as a function of pipe diameter, viscosity (η), and particle density (ρ_s). Dashed line represents the maximum fluid velocity that can be used safely in an industrial system. Green shaded region shows safe operating conditions for porous liquid 1. Red shaded region shows inoperable velocities for liquid 1 due to particle accumulation and/or unsafe velocities.

The critical deposition velocities of various MOFs in several fluids are shown in **Figure S4** to represent several dispersions (some of which are Type III porous liquids). When interpreting the following results, it is critical to understand that these calculations are only valid for qualitative comparisons between different slurries. The critical deposition velocity results shown in **Figure**

S4 are not necessarily representative of the actual critical deposition velocity of the mentioned slurries because of a lack of information on interactions between the solvent and the porous solids, especially for nanoscopic particles such as those typically used in Type III porous liquids. Type III porous liquids have potential in gas separations and various other applications. Still, researchers must consider the impact of the critical deposition velocity on those materials in industrial use.

Table S2: MOF Densities[3-10]

MOF	Density (g/cm^3)	Reference
HKUST-1	1.03	3
ZIF-7	1.39	4
UiO-66	1.44	5
MOF-801	1.59	6
ZIF-67	1.63	7
Cu(Qc)2	1.50	8
SIFSIX-3-Cu	1.60	9
Zn MOF 74	1.92	10

The MOFs were chosen to get a range of densities for the critical deposition velocity analysis. More information on the chosen MOFs can be found in the supplemental information (**Table S2**). Water and methanol do not create porous liquids with any of the MOFs used because they are small enough to penetrate the pores of the MOFs, but they serve as good lower-bound solvents for this analysis because of their low viscosity. 2'-hydroxyacetophenone, as explained previously, has been used to create Type II porous liquids and has a moderate viscosity of 3.9 cP at ambient conditions. Sesame oil was chosen as an upper bound solvent since it has the highest viscosity and has been referenced as an effective and inexpensive solvent for creating Type III porous liquids. The viscosities of methanol, water, 2HAP, and sesame oil employed were 0.7, 1.0, 3.9, and 31 cP, respectively.

For each carrier fluid and MOF combination, the deposition velocity follows similar trends when plotted against the pipe diameter. For the low viscosity carrier fluids, water and methanol, the deposition velocities range from 4-12 m/s at a pipe diameter of 0.05 m and 5-21 m/s at a pipe diameter of 0.3 m. 2'-hydroxyacetophenone has a viscosity of about 4 times that of water and leads to a critical deposition velocity increase of about 10-15% compared to the water/MOF combinations. Lastly, sesame oil is an order of magnitude more viscous than 2'-hydroxyacetophenone. This large increase in viscosity leads to a much larger set of critical deposition velocities that can exceed 45 m/s for the densest MOFs (Zn MOF-74). $Cu(Qc)_2$ dispersed in sesame oil creates a Type III porous liquid that shows promise for ethane/ethylene separations; however, that dispersion has an estimated critical deposition velocity ranging from 25-38 m/s. Although sesame oil is a cheap and effective material for making a porous liquid, it could lead to porous liquids with quite high deposition velocities, which may limit its use in gas

separation applications. As stated in the main text these calculations are not representative of actual critical deposition velocities of these slurries due to lack of experimental information. But they are relevant for qualitative comparison.

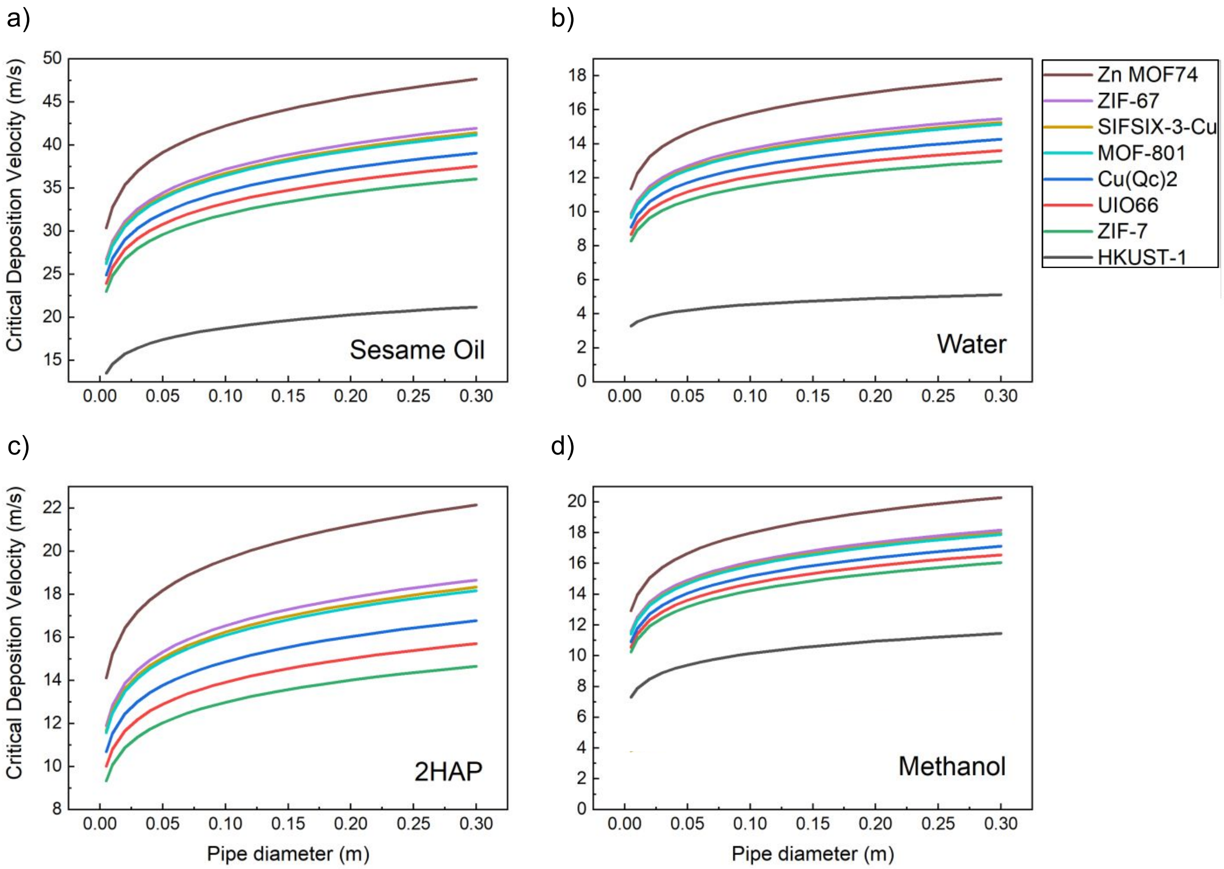

Figure S4: Critical deposition velocity calculations for various MOFs and POCs dispersed in a) sesame oil, b) water, c) 2'-hydroxyacetophenone, and d) methanol as a function of pipe diameter.

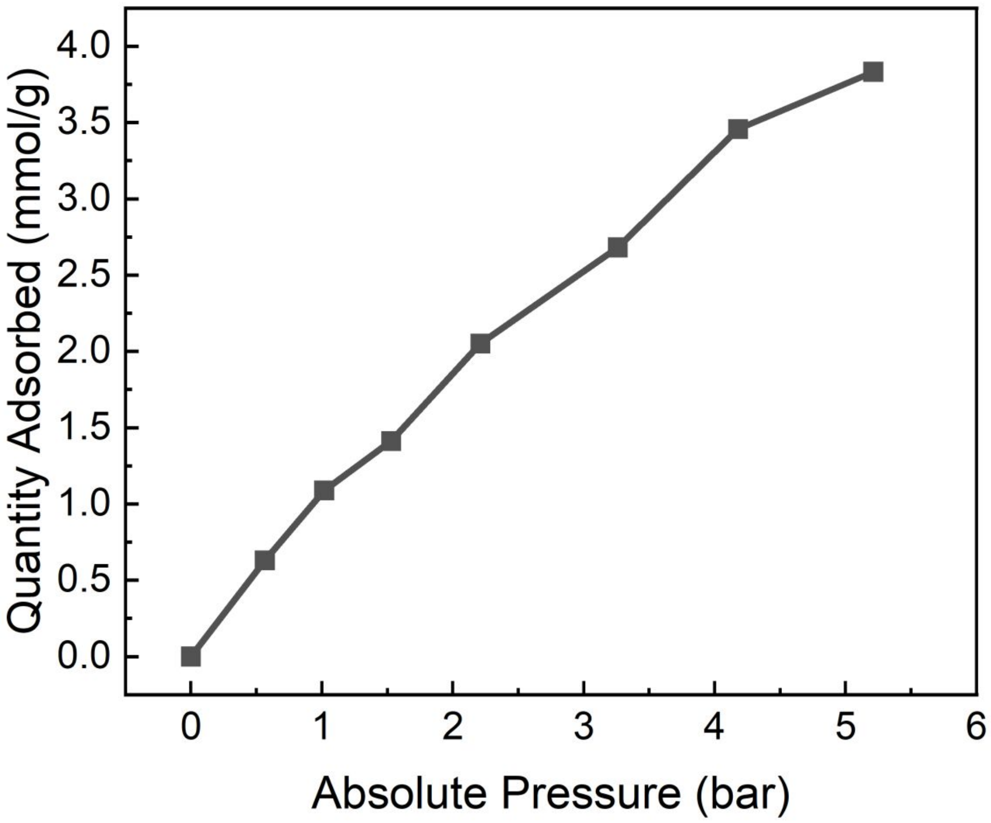

Figure S5: CO_2 isotherm at 30 °C for CC13

Table S3: Raw Data CO_2 Isotherm on CC13 at 30 °C

Absolute Pressure (bar)	Quantity Adsorbed (mmol/g)
0	0
0.56	0.63
1.02	1.09
1.53	1.41
2.21	2.05
3.26	2.68
4.18	3.46
5.21	3.83

Table S4: Pure Solvent CO_2 Isotherms at 30 °C

2HAP		2CP		2IPP	
Pressure (bar)	Quantity Absorbed (mmol/g)	Pressure (bar)	Quantity Absorbed (mmol/g)	Pressure (bar)	Quantity Absorbed (mmol/g)
0.63	0.12	0.63	0.09	0.59	0.13
1.22	0.24	1.43	0.19	1.17	0.27
1.69	0.32	2.39	0.35	2.23	0.46
2.18	0.42	3.23	0.48	3.17	0.57
3.11	0.67	4.72	0.67	4.07	0.71
4.07	0.82			5.09	0.82
5.07	1.09				

Table S5: Porous Liquid CO_2 Isotherms at 30 °C

2HAP		2CP		2IPP	
Pressure (bar)	Quantity Absorbed (mmol/g)	Pressure (bar)	Quantity Absorbed (mmol/g)	Pressure (bar)	Quantity Absorbed (mmol/g)
0.59	0.17	0.57	0.14	0.57	0.21
1.31	0.38	1.21	0.36	1.07	0.42
2.01	0.59	1.98	0.54	1.83	0.68
2.94	0.92	3.07	0.77	2.83	1.02
3.89	1.26	4.02	1.03	3.84	1.25
4.89	1.62	5.01	1.25	4.87	1.54

References

1. Rice HP, Fairweather M, Peakall J, Hunter TN, Mahmoud B, Biggs SR. Constraints on the Functional Form of the Critical Deposition Velocity in Solid–Liquid Pipe Flow at Low Solid Volume Fractions. Chemical Engineering Science. 2015;126:759-770.
2. Bbosa B, Dellecase E, Volk M, Ozbayoglu E. A Comprehensive Deposition Velocity Model for Slurry Transport in Horizontal Pipelines. Journal of Petroleum Exploration and Production Technology. 2017;7(1):303-310.
3. Yakovenko AA, Reibenspies JH, Bhuvanesh N, Zhou HC. Generation and applications of structure envelopes for porous metal-organic frameworks. J. Appl. Cryst. 2013;46:346-353.
4. Cai W, Lee T, Lee, M, Cho W, H DY, Choi N, Yip ACK, Choi J. Thermal Structural Transitions and Carbon Dioxide Adsorption Properties of Zeolitic Imidazolate Framework-7 (ZIF-7). J. Am. Chem. Soc. 2014;136(22):7961–7971.
5. Øien S, Wragg D, Reinsch H, Svelle S, Bordiga S, Lamberti C, Lillerud KP. Detailed Structure Analysis of Atomic Positions and Defects in Zirconium Metal–Organic Frameworks. Cryst. Growth Des. 2014;14(11):5370–5372.
6. Furukawa H, Gándara F., Zhang YB, Jiang J, Queen WL, Hudson MR, Yaghi OM. Water Adsorption in Porous Metal–Organic Frameworks and Related Materials. J. Am. Chem. Soc. 2014;136(11):4369–4381.
7. Kwon HT, Jeong HK, Lee AS, An HS, Lee JS. Heteroepitaxially Grown Zeolitic Imidazolate Framework Membranes with Unprecedented Propylene/Propane Separation Performances. J. Am. Chem. Soc. 2015;137(38):12304–12311.
8. Lin RB, Wu H, Tang XL, Li Z, Gao J, Cui H, Zhou W, Bhen B. Boosting Ethane/Ethylene Separation within Isoreticular Ultramicroporous Metal–Organic Frameworks. J. Am. Chem. Soc. 2018;140(40):12940–12946.
9. Shekhah O, Belmabkhout Y, Chen Z, Guillerm V, Cairns A, Adil K, Eddaoudi M. Made-to-order metal-organic frameworks for trace carbon dioxide removal and air capture. Nature Communications. 2014;5:4228.
10. Rosi NL, Kim J, Eddaoudi M, Chen B, O'Keeffe M, Yaghi OM. Rod Packings and Metal−Organic Frameworks Constructed from Rod-Shaped Secondary Building Units. J. Am. Chem. Soc. 2005;127(5):1504–1518.
11. Salama MM, Venkatesh ES. Evaluation of API RP 14E Erosional Velocity Limitations for Offshore Gas Wells. Offshore Technology Conference 1983. OnePetro.
12. API RP 14E, Recommended Practice for Design and Installation of Offshore Production Platform Piping Systems. Institute, A. P., Ed. 1991; Vol. 5th ed.
